# Complex analysis of the personalized pharmacotherapy in the management of COVID-19 patients and suggestions for applications of predictive, preventive, and personalized medicine attitude

**DOI:** 10.1007/s13167-021-00247-0

**Published:** 2021-07-16

**Authors:** Lei-Yun Wang, Jia-Jia Cui, Qian-Ying OuYang, Yan Zhan, Yi-Min Wang, Xiang-Yang Xu, Lu-Lu Yu, Hui Yin, Yang Wang, Chen-Hui Luo, Cheng-Xian Guo, Ji-Ye Yin

**Affiliations:** 1 Department of Clinical Pharmacology, Xiangya Hospital, Central South University, Changsha, 410078 Hunan People’s Republic of China; 2 Institute of Clinical Pharmacology, Central South University, Changsha, China; 3 Hunan Key Laboratory of Pharmacogenetics, Changsha, 410078 Hunan People’s Republic of China; 4 Engineering Research Center of Applied Technology of Pharmacogenomics, Ministry of Education, 110 Xiangya Road, Changsha, 410078 Hunan People’s Republic of China; 5 National Clinical Research Center for Geriatric Disorders, 87 Xiangya Road, Changsha, 410008 Hunan People’s Republic of China; 6 Genetalks Co., Ltd., Changsha, 410008 Hunan People’s Republic of China; 7 Scientific Research Office, Hunan Cancer Hospital, the Affiliated Cancer Hospital of Xiangya School of Medicine, Central South University, Changsha, 410013 Hunan People’s Republic of China; 8 Center of Clinical Pharmacology, the Third Xiangya Hospital, Central South University, Changsha, 410013 Hunan People’s Republic of China; 9 Hunan Key Laboratory of Precise Diagnosis and Treatment of Gastrointestinal Tumor, Changsha, 410078 Hunan People’s Republic of China

**Keywords:** COVID-19, Predictive preventive personalized medicine (PPPM/3 PM), Drug-to-drug interaction, Pharmacogenomics, Ethnicity-based differences, Future healthcare, Gene score, Drug score, Optimal medication, Personalized treatment, Individual outcomes, Molecular mechanisms, Pharmacogenetics, Comorbidities, Ritonavir, Daclatasvir, Sofosbuvir, Ribavirin, Interferon alpha-2b, Chloroquine, Hydroxychloroquine, Ceftriaxone, Ritonavir, Daclatasvir, Prednisone, Dexamethasone, Ribavirin, HCQ, Ceftriaxone, Zinc, Interferon beta-1a, Remdesivir, Levofloxacin, Lopinavir, Human immunoglobulin G, Losartan

## Abstract

**Aims:**

Coronavirus disease 2019 (COVID-19) is rapidly spreading worldwide. Drug therapy is one of the major treatments, but contradictory results of clinical trials have been reported among different individuals. Furthermore, comprehensive analysis of personalized pharmacotherapy is still lacking. In this study, analyses were performed on 47 well-characterized COVID-19 drugs used in the personalized treatment of COVID-19.

**Methods:**

Clinical trials with published results of drugs use for COVID-19 treatment were collected to evaluate drug efficacy. Drug-to-Drug Interactions (DDIs) were summarized and classified. Functional variations in actionable pharmacogenes were collected and systematically analysed. “Gene Score” and “Drug Score” were defined and calculated to systematically analyse ethnicity-based genetic differences, which are important for the safer use of COVID-19 drugs.

**Results:**

Our results indicated that four antiviral agents (ritonavir, darunavir, daclatasvir and sofosbuvir) and three immune regulators (budesonide, colchicine and prednisone) as well as heparin and enalapril could generate the highest number of DDIs with common concomitantly utilized drugs. Eight drugs (ritonavir, daclatasvir, sofosbuvir, ribavirin, interferon alpha-2b, chloroquine, hydroxychloroquine (HCQ) and ceftriaxone had actionable pharmacogenomics (PGx) biomarkers among all ethnic groups. Fourteen drugs (ritonavir, daclatasvir, prednisone, dexamethasone, ribavirin, HCQ, ceftriaxone, zinc, interferon beta-1a, remdesivir, levofloxacin, lopinavir, human immunoglobulin G and losartan) showed significantly different pharmacogenomic characteristics in relation to the ethnic origin of the patient.

**Conclusion:**

We recommend that particularly for patients with comorbidities to avoid serious DDIs, the predictive, preventive, and personalized medicine (PPPM, 3 PM) strategies have to be applied for COVID-19 treatment, and genetic tests should be performed for drugs with actionable pharmacogenes, especially in some ethnic groups with a higher frequency of functional variations, as our analysis showed. We also suggest that drugs associated with higher ethnic genetic differences should be given priority in future pharmacogenetic studies for COVID-19 management. To facilitate translation of our results into clinical practice, an approach conform with PPPM/3 PM principles was suggested. In summary, the proposed PPPM/3 PM attitude should be obligatory considered for the overall COVID-19 management.

**Supplementary Information:**

The online version contains supplementary material available at 10.1007/s13167-021-00247-0.

## Introduction

### COVID-19

Last year, 2020, the World Health Organization (WHO) reported a new disease caused by a novel coronavirus named severe acute respiratory syndrome coronavirus 2 (SARS-CoV-2) or COVID-19 and declared a Public Health Emergency of International Concern [1–4]. Currently, COVID-19 is experiencing rapid outbreak. A large number of patients have been infected and died due to this disease (https://coronavirus.jhu.edu/map.html). The virus is mainly spread by respiratory droplets. The most common clinical features of COVID-19 are fever, dry cough and shortness of breath, accompanied by particular abnormalities detected in clinical laboratories, such as lymphopenia and elevated lactate dehydrogenase, as well as the results of in vivo imaging procedures [5]. Millions of COVID-19 cases could progress rapidly to acute respiratory distress syndrome (ARDS), shock, multiple organ failure and even death [6–8]. Therefore, a large number of patients needed to be appropriately treated without delay in this situation.

### State of the art in COVID-19 treatment

The current management for COVID-19 mainly includes antiviral treatment and symptomatic treatment. The former involves antiviral drugs, neutralizing antibodies and convalescent plasma from COVID-19 patients, and the latter consists of drug therapy and multiorgan functional support. Of these, drug therapy is an important approach to fight against COVID-19. The WHO COVID-19 Living Clinical Management Guidance suggested that in addition to standard care, antiviral agents, immunomodulators and other drugs were also recommended. The results of existing clinical trials show that these drugs may benefit patients with COVID-19, and a large number of patients are receiving drug treatments in the clinic. However, based on the results from current clinical trials, several unsatisfactory results were observed in the utilization of some drugs. Certain patients may not necessarily benefit from drug therapy or even suffer from side effects, especially those with comorbidities or from certain ethnic groups. These phenomena imply individual differences and suggest the importance of (predictive, preventive, and personalized medicine) PPPM for COVID-19. [9].

### Drug-to-drug interactions

As mentioned above, Drug-to-Drug Interactions (DDI) may be a factor in unsatisfactory outcomes in patients receiving multidrug therapy. DDIs are mainly involved in altering the blood concentration of drugs when they are used with other drugs concomitantly, by changing the metabolic rate or the drug excretion, and increasing side effects. Patients undergoing the combined use of multiple drugs are more likely to suffer from potential DDIs [10, 11]. For example, a cross-sectional study found that antiarrhythmic drugs, symvastatin and budesonide are the main substrates of *CYP3A* and are often used in combination with the commonly used COVID-19 drug lopinavir-ritonavir (LPV-r), which may increase the concentration of these drugs and may be related to life-threatening reactions [12]. Therefore, it is important to prevent DDIs by not using particular drugs in risky combinations during COVID-19 management.

### Pharmacogenes

It is widely accepted that genetic factor is one of the major contributors to individual and ethnicity-based differences related to drug therapeutic efficacy and toxicity [13–17]. At the same time, pharmacogenomic studies (PGx) have revealed that the association between different genetic backgrounds and differ the drug effects seen in different patients may be another factor to explain the individual differences [18, 19]. PGx studies have focused on the variations across the genome that could lead to differences in drug responses. The genes in which these variations are located are called “pharmacogenes”. Pharmacogenes are generally involved in the process of pharmacokinetics or pharmacodynamics. The former mainly includes the absorption, distribution, metabolism and excretion of drugs in the body, while the latter mainly involves the effects of drugs on the body and their mechanism. Accordingly, pharmacogenes are divided into four types, including drug targets, transporters, metabolizing enzymes and some other genes. Variations in pharmacogenes may affect the gene function, which in turn may influence the efficiency or toxicity of drugs. Genetic testing for these variations may be used to distinguish populations with potentially unfavourable drug responses. Some of pharmacogenes have been utilized to guide PPPM/3 PM in clinical practice and are called actionable pharmacogenes. For example, variants that decrease *CYP2C9* activity are associated with an increase in warfarin plasma concentration and bleeding risk. However, most drugs do not have a corresponding pharmacogene to predict their drug response or toxicity. Therefore, more studies should be launched to find more actionable pharmacogenes.

### Role of ethnicity

**Fig. 1:**
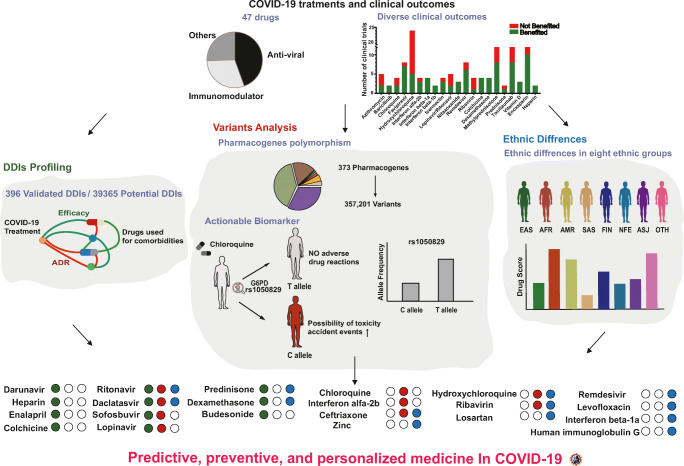
**Overview of individual disparities indicating PPPM for COVID-19.** Diagrams of the sources of data and analyses performed for predictive, preventive, and personalized COVID-19 treatment. The three parts in the middle of this figure indicate the different analyses utilized in this study. In the bottom panel, the final list of 22 drugs that showed high individual disparity was presented, and the green circle, red circle and blue circle indicate DDI analyses, actionable pharmacogene analyses and ethnic difference analyses, respectively. The solid circle to the right side of the drug name indicates that this drug needed PPPM-attitude-based management in the area corresponding to the colour of the circle, while a hollow circle indicates management was not needed. For example, with ritonavir, the colour code green, colour code red and colour code bule indicate that DDIs, actionable pharmacogenes and ethnic differences should be considered for this drug in COVID-19 treatment, while for ribavirin the colour code empty green, colour code red and colour code blue indicate that both actionable pharmacogenes and ethnic differences rather than DDIs, should be considered

Based on the similar genetic background of the same ethnic group, the frequency of gene variation may be specific to ethnicity among different populations. At the same time, we observed in the results of existing clinical trials that the drug response to COVID-19 varies among different ethnic groups. For example, a significant difference was observed in hydroxychloroquine (HCQ) efficacy between non-Finnish European (NFE) and East Asian (EAS) patients in clinical trials, and EAS patients tended to have a higher drug response than NFE patients. In contrast, for remdesivir, NFE patients showed better efficacy than EAS with treatment. The interethnic differences in drug efficacy may be associated with the ethnic differences in pharmacogenes. Therefore, studying ethnic differences in pharmacogene variations may facilitate the PPPM attitude towards the treatment of COVID-19. Moreover, studies reported that certain factors other than ethnicity, such as blood platelet counts, could be used to determine the prognosis of COVID-19 treatment [20]. However, PPPM for drug utilization in COVID-19 treatment still seems to be ignored. In the current study, we conducted a comprehensive personalized treatment analysis of 47 well-characterized drugs used for COVID-19 treatment (Fig. 1).

## Materials and methods

### Data collection

Forty-seven well-characterized drugs used for COVID-19 treatment and 132 related clinical trial results (Table S1) were collected from publications (PubMed: https://pubmed.ncbi.nlm.nih.gov/; MedRxiv: https://www.medrxiv.org/; BioRxiv: https://www.biorxiv.org/; Cochrane Library: https://www.cochranelibrary.com/;) and ClinicalTrials (https://www.clinicaltrials.gov/). Information on 397 validated DDIs was retrieved from the FDA (https://www.fda.gov/) and 39,365 predicted DDIs were derived from DrugBank (https://www.drugbank.ca/).

The 373 pharmacogenes related to all 47 drugs were obtained from the PharmGKB (https://www.pharmgkb.org/) and DrugBank (https://www.drugbank.ca/) databases. Genetic variation data for each pharmacogene were retrieved from the Genome Aggregation Database (http://gnomAD.broadinstitute.org/, version: 2.1.1) [21]. Based on the analysis of 125,748 subjects, 357,201 variants were retrieved in total (Table S6). Actionable PGx biomarkers were gathered from the FDA (https://www.fda.gov/drugs/science-and-research-drugs/table-pharmacogenomic-biomarkers-drug-labelling) and CPIC guidelines (https://cpicpgx.org/guidelines/), which contained information about changes in efficacy, dosage, metabolism or toxicity due to genetic variants or phenotypes.

### Drug-to-drug interaction analysis

The effects on efficacy and adverse drug reactions caused by drug-to-drug interactions were determined on the basis of FDA drug labels. The classification of drugs was derived from the ATC codes (https://www.whocc.no/atc_ddd_index/). The drug-to-drug interaction network was constructed by Cytoscape software (version:3.7.1) [22].

### Annotation of genetic variations

All genetic variations were annotated by allele frequency, location and function in different populations using ANNOVAR (version: 2019Oct24) [23]. In the current study, populations were divided into eight categories, on the basis of ethnic origin as described previously: African ethnic origin (AFR), Latino ethnic origin (AMR), EAS, South Asian ethnic origin (SAS), Finnish ethnic origin (FIN), non-Finnish ethnic origin (NFE), Ashkenazi Jewish ethnic origin (ASJ) and Other ethnic origin (OTH) [21]. Based on the location and functions, all mutations were divided into 7 categories: frameshift, nonframeshift, synonymous, nonsynonymous, untranslated regions, intronic and others (including splicing, upstream of a gene, downstream of a gene, stop-gain, stop-loss, ncRNA-exonic and unknown mutations). Reported functional variations were collected from CPIC guidelines (https://cpicpgx.org/guidelines/) and PharmGKB (https://www.pharmgkb.org/). Functional mutations were predicted by PROVEAN and SIFT [24].

### Definition and computation of “gene score” and “drug score”

For the purpose of evaluating the carrying levels of homozygous deleterious alleles in COVID-19 patients, we developed the “Gene Score”, which represents the homozygous rate of damage variants in patients. The algorithm was derived from a previous study [25]. We enrolled all potential damage variants that were predicted by PROVEAN and SIFT to calculate the “Gene Score”. This score provided a probability that a person is carrying the homozygous deleterious allele “*a*” in a specific gene:

$$ Gene\ Score=1-\prod \limits_{a\in DV}\left(1- AF{(a)}^2\right) $$

Damage variants (“DV”) were set as a collection of all predicted damage variants of a specific gene, while “*a*” presented each variant in the “DV” set. Allele frequency (“AF”) indicates the frequency of the damage variants.

The parameter “Gene Score” indicated that if the frequency of carrying at least a homozygous potential deleterious mutation in a pharmacogene is higher in an ethnic group than in the other groups, the “Gene Score” of this pharmacogene is higher in this ethnic group. Therefore, abnormal function of this pharmacogene is more likely in this ethnic group, and a pharmacogenetic study of this pharmacogene is encouraged.

The degree of ethnicity-based differences of a drug related to homozygous deleterious variants led us to define the “Drug Score”, which is integrated with all “Gene Scores” of drug- relevant pharmacogenes. Therefore, the calculation formula is as follow:

$$ {Drug\ Score}_{(A)}={\varSigma}_{g\in DG}\left({\mathrm{Gene}\ \mathrm{Score}}_{g(A)}-\frac{1}{7}\ast {\sum}_1^7\left({\mathrm{Gene}\ \mathrm{Score}}_{g(other)}\ \right)\right) $$

Drug related pharmacogenes (“DG”) are a collection of all related drug pharmacogenes of a specific drug, while “*g*” represents each pharmacogene in the “DG” set. Ethnicity “A” arbitrarily indicates one ethnicity among AFR, AMR, EAS, SAS, FIN, NFE, ASJ and OTH, and “Gene Score_*g*(*A*)_” indicates the “Gene Score” of the pharmacogene “*g*” in ethnicity “A”. The formula “$\frac{1}{7}\ast {\sum}_1^7\left({\mathrm{Gene}\ \mathrm{Score}}_{g(other)}\ \right)$” indicates the average “Gene Score” of the pharmacogene “*g*” in seven other ethnicities except ethnicity “A”.

The parameter “Drug score” indicates the difference in the cumulative disparity of all known pharmacogenes of a drug from an ethnic group to seven other ethnic groups which were described in this study. A higher “Drug score” indicates a greater genetic diversity of a drug among ethnic groups. Therefore, pharmacogenetic study of this drug is encouraged.

### Statistical analysis and data visualization

Statistical analysis was performed in SPSS software (version: 18.0), and results with *p* < 0.05 were considered statistically significant. Data visualization was performed by GraphPad Prism software (version: 8.0.2).

## Results

To solve the problems we referred above, we performed a complex analysis of the personalized pharmacotherapy. The results presented below we are very important for practical application of the principles of predictive, preventive and personalized medicine in the clinical practice of COVID-19 management. For better clarity we try to give the particular examples (Tables 1, 2 and 3) based on our findings.

**Table 1:** Top 5 DDIs in COVID-19 treatment. For the extended list of description of other DDIs, please, see Supplementary Table S2

Drug1^a^	Drug2^a^	DDI description^b^	DDI Category^c^	Number of DDI in FDA^d^	Number of DDI in Drugbank^d^
Ritonavir				119	1483
	Ranolazine	The serum concentration of Ranolazine can be increased when it is combined with Ritonavir.	Efficacy		
	Disulfiram	The risk or severity of adverse effects can be increased when Ritonavir is combined with Disulfiram.	Side effects		
Darunavir				119	582
	Lovastatin	The serum concentration of Lovastatin can be increased when it is combined with Darunavir.	Efficacy		
	Buspirone	The risk or severity of adverse effects can be increased when Darunavir is combined with buspirone.	Side effects		
Daclatasvir				19	478
	Digoxin	Daclatasvir may decrease the excretion rate of Digoxin which could result in a higher serum level.	Efficacy		
	Amiodarone	Daclatasvir may increase the bradycardic activities of Amiodarone.	Side effects		
Heparin				18	898
	Nitroglycerin	Nitroglycerin may decrease the anticoagulant activities of Heparin.	Efficacy		
	Warfarin	The risk or severity of bleeding can be increased when Warfarin is combined with Heparin.	Side effects		
Prednisone				13	1464
	Cholestyramine	Cholestyramine may increase the excretion rate of Prednisone which could result in a lower serum level and potentially a reduction in efficacy.	Efficacy		
	Amphotericin B	The risk or severity of hypokalemia can be increased when Prednisone is combined with Amphotericin B.	Side effects		

a: “Drug1” indicates that these drugs were utilized for COVID-19 treatment, and “Drug2” indicates that these drugs were utilized for basic comorbidities

b: The impacts of DDIs on patients were described in detail

c: All DDIs were classified into two classes. If the DDI was reported to generate potential side effects, this DDI was tagged as “Side effects” in this column. Other DDIs which may impact the concentration or the effects of drugs were tagged as “Efficacy” in this column

d: The total number of DDIs of this drug in FDA or Drugbank was presented

**Table 2:** “Gene Scores” of pharmacogenes for COVID-19 treatment

Gene type	Gene name	Gene Score^e^							
		AFR^f^	SAS^f^	AME^f^	EAS^f^	NFE^f^	FIN^f^	ASJ^f^	OTH^f^
Transporters^a^									
	SLC47A1^g^	0.003	0.003	0.019	0.003	0.003	0.003	0.003	0.003
	Mean score^h^	0.025	0.015	0.016	0.029	0.005	0.010	0.009	0.004
Metabolizing enzyme genes^b^									
	G6PD^g^	0.013	0.001	0.002	0.002	0.002	0.002	0.002	0.002
	Mean score^h^	0.014	0.006	0.009	0.019	0.005	0.006	0.007	0.002
Targeted genes^c^									
	VDR^g^	0.239	0.202	0.155	0.134	0.033	0.010	0.106	0.003
	Mean score^h^	0.012	0.008	0.009	0.013	0.006	0.006	0.009	0.004
Other genes^d^									
	IFNL3^g^	0.002	0.001	0.004	0.005	0.001	0.001	0.001	0.001
	Mean score^h^	0.020	0.009	0.011	0.016	0.007	0.012	0.008	0.005

a: “Transporters are pharmacogenes” which can transport drugs or metabolites of drugs into or out of cells. The abnormal function of transporters can affect the concentration of drugs

b: “Metabolizing enzyme genes” are pharmacogenes which can metabolize drugs into active metabolites or inactive metabolites. The abnormal function of metabolizing enzymes can affect the effects of drugs through increasing or decreasing the metabolizing rate

c: “Targeted genes” are the direct targets of drugs. The abnormal function of targets can directly affect the binding affinity of drugs, thus largely impact on the effects of drugs

d: “Other genes” can affect the effects of drugs but not belong to these three categories of pharmacogenes mentioned above. These pharmacogenes may be involved in the pathways of drugs’ effects (eg. IFNL3), or impact on the efficacy of drugs through changing the plasma protein binding rate (eg. ALB)

e: “Gene Score” indicates the frequency of carrying at least a homozygous potential deleterious mutation in this pharmacogene. A higher “Gene Score” indicates that abnormal function of this pharmacogene could be more likely found in this ethnic group

f: The calculated “Gene Score” in eight ethnic groups including: AFR-African, AMR-Latino, EAS-East Asian, SAS-South Asian, FIN-Finnish, NFE-non-Finnish European, ASJ-Ashkenazi Jewish, OTH-Other

g: The pharmacogenes of drugs in COVID-19 treatment including: SLC47A1-Solute carrier family 47 member 1, G6PD-Glucose-6-phosphate dehydrogenase, VDR-Vitamin D receptor, IFNL3-Interferon lambda 3

h: The mean “Gene Score” of all pharmacogenes in the each category was calculated for eight ethnic groups

**Table 3:** Ethnicity based genetic difference in medicine for COVID-19 treatment

Drug name	Drug Score^a^								Ethnicity with a “Drug Score” over 0.05
	AFR^b^	SAS^b^	AMR^b^	EAS^b^	NFE^b^	FIN^b^	ASJ^b^	OTH^b^	
Zinc	0.798	0.322	0.435	0.530	0.231	0.314	0.249	0.187	AFR, SAS, AMR, EAS, NFE, FIN, ASJ, OTH
Prednisone	0.369	0.120	0.092	0.369	0.098	0.145	0.146	0.070	AFR, SAS, AMR, EAS, NFE, FIN, ASJ, OTH
Ribavirin	0.161	0.174	0.138	0.243	0.154	0.152	0.213	0.077	AFR, SAS, AMR, EAS, NFE, FIN, ASJ, OTH
Dexamethasone	0.247	0.115	0.148	0.192	0.042	0.050	0.124	0.028	AFR, SAS, AMR, EAS, ASJ
Ritonavir	0.214	0.103	0.096	0.275	0.042	0.109	0.035	0.048	AFR, SAS, AMR, EAS, FIN
Interferon beta-1a	0.094	0.103	0.136	0.143	0.052	0.069	0.155	0.038	AFR, SAS, AMR, EAS, NFE, FIN, ASJ
Remdesivir	0.091	0.082	0.074	0.214	0.046	0.092	0.060	0.024	AFR, SAS, AMR, EAS, FIN, ASJ
Levofloxacin	0.137	0.096	0.121	0.115	0.032	0.080	0.051	0.012	AFR, SAS, AMR, EAS, FIN, ASJ
Daclatasvir	0.146	0.095	0.073	0.074	0.027	0.022	0.063	0.014	AFR, SAS, AMR, EAS, ASJ
Lopinavir	0.086	0.044	0.045	0.136	0.020	0.048	0.031	0.024	AFR, EAS
Human immunoglobulin G	0.093	0.048	0.054	0.074	0.039	0.054	0.042	0.025	AFR, AMR, EAS, FIN
Ceftriaxone	0.062	0.053	0.066	0.150	0.013	0.019	0.030	0.025	AFR, SAS, AMR, EAS
Losartan	0.142	0.059	0.012	0.126	0.011	0.008	0.032	0.016	AFR, SAS, EAS
Hydroxychloroquine	0.087	0.054	0.039	0.135	0.013	0.038	0.017	0.021	AFR, SAS, EAS

a: “Drug Score” indicates the difference of the cumulative disparity of all known pharmacogenes of a drug from an ethnic group to all other 7 ethnic groups. A higher “Drug score” indicates a greater genetic diversity of a drug among ethnic groups

b: The details of calculated “Drug Score” in Fig. 6C, D and E. AFR-African, AMR-Latino, EAS-East Asian, SAS-South Asian, FIN-Finnish, NFE-non-Finnish European, ASJ-Ashkenazi Jewish, OTH-Other

### Diverse clinical outcomes of drugs in COVID-19 treatment

**Fig. 2:**
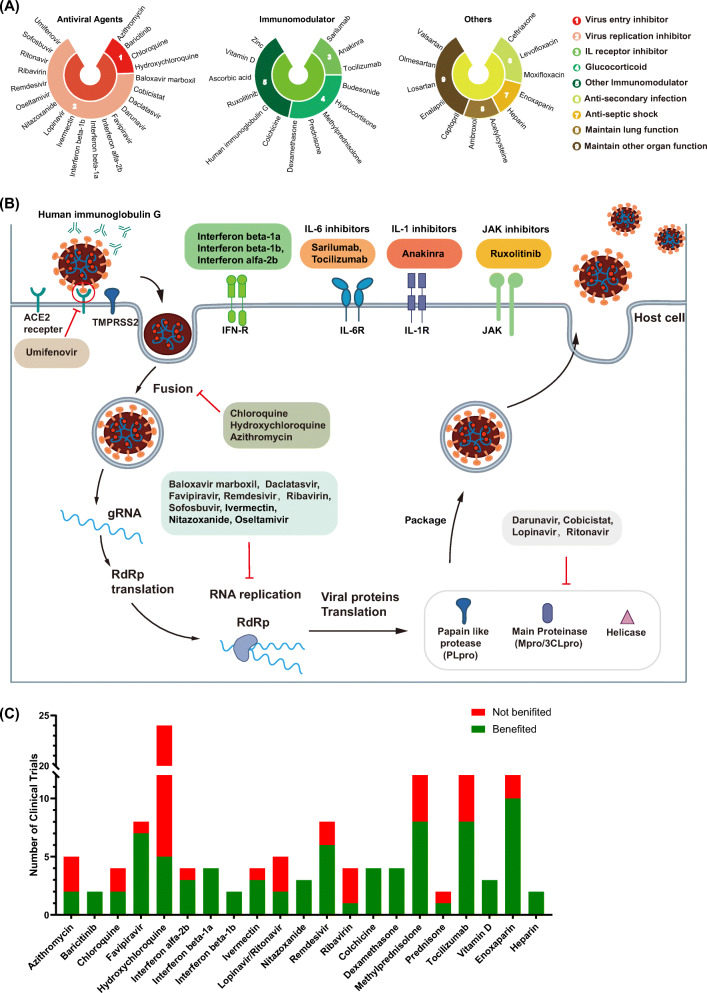
**Drugs for COVID-19 therapy.** (A) All 47 drugs utilized for COVID-19 therapy were divided into three categories and nine subcategories are depicted. The categories of drugs are indicated by different colours, as presented in the figure legend. (B) The whole process of virus invasion is depicted. Drugs effects in each step are listed to the side. (C) The outcomes of clinical trials of drugs for COVID-19 treatment. Green indicates that patients benefited in the clinical trial compared to the control group, while red indicates that patients did not benefit. The Y-axis indicates the number of clinical trials

We systematically searched published studies and clinical trials with results from COVID-19 treatment (Table S1). Forty-seven well-characterized drugs, which could be classified into three categories as antiviral agents, immunoregulatory agents and drugs with other important functions in constraining COVID-19, were enrolled in this study (Fig. 2A) [26, 27]. Among them, antiviral drugs included virus entry inhibitors, virus replication inhibitors and other antiviral agents. These antiviral drugs that participate in many aspects of the virus life cycle, have been utilized since the early stage of confirmed COVID-19 infection. As shown in Fig. 2B, inhibitors targeting viral protease accounted for the majority of these drugs, followed by inhibitors that target RNA-dependent RNA polymerase (RdRp). Moreover, pneumonia-related symptoms were observed in many patients. These symptoms were mainly induced by abnormal inflammatory function. Therefore, both anti-inflammatory agents and immunomodulatory drugs could be utilized to alleviate these symptoms. Drugs for other purposes such as anti-secondary infection agents and lung-function-maintaining agents were also widely utilized to prevent patients from experiencing additional suffering. It should be noticed that contradictory results were widely observed in some trials, which indicated potential individual differences and a demand for personalized COVID-19 treatment (Fig. 2C). There were 132 results reported in clinical trials of a total number of 21 drugs. Among them, 13 drugs reached diverse conclusions. The beneficial and nonbeneficial clinical outcomes of azithromycin, chloroquine, lopinavir and ritonavir were near 50%, respectively.

These diverse clinical outcomes further indicated potential individual disparity in COVID-19 treatment, and factors that potentially impacted the clinical outcomes of individuals should be considered in PPPM/3 PM for COVID-19. We then performed systematic analyses for this purpose.

### Profiling of potential DDIs

In addition to the primary infections, many studies have indicated that large numbers of severe COVID-19 patients are diagnosed with other comorbidities, such as psychiatric diseases, cardiovascular diseases or hypertensions [28–30]. They were reported to utilize multiple drugs simultaneously during the treatment. Therefore, potential DDIs may be generated. We systematically screened 39,761 pairs of predicted DDIs for all 47 drugs in our study (Table S2) [31]. Examples of the top 5 most interacting drugs are provided (Table 1). All of these DDIs were found to potentially affect drug efficacy or side effects. They indicated that patients with multiple symptoms should be treated more carefully.

**Fig. 3:**
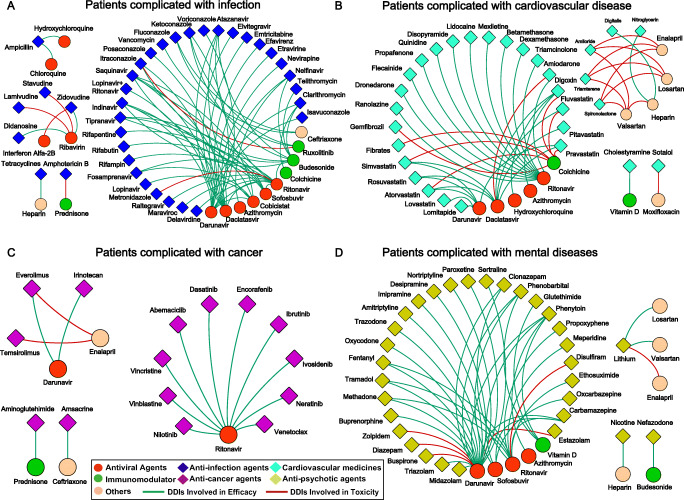
**Drug-to-Drug interactions profiling by the FDA.** Drug-to-drug interactions determined by the FDA labelled between COVID-19 treatment drugs and (A) anti-infection agents, (B) cardiovascular medicines, (C) anticancer agents, (D) antipsychotic agents. According to the legends, the circles indicate drugs in the COVID-19 regimens, while the diamonds indicate drugs in the basic regimens. Among them, the red circles, green circles and the orange circles are antiviral agents, immunomodulators and other drugs in the COVID-19 regimens respectively. Similarly, dark blue diamonds, indigo blue diamonds, violet diamonds and yellow diamonds represent anti-infection agents, cardiovascular drugs, anticancer agents and anti-psychotic agents respectively. If there is a DDI between two drugs, these two drugs are linked by a line. The red line indicates that this DDI can generate toxicity, while the green line indicates that this DDI can impact the efficacy by increasing or decreasing the concentration as listed in Table S2

To make this result more credible, we retrieved 397 validated DDIs in the clinic for all 47 drugs. These DDIs should receive more attention. There were four major categories of the most common concomitantly utilized drugs during COVID-19 treatment, including anti-infective agents, cardiovascular drugs, antitumor agents and antipsychotics (Fig. 3). As indicated, we could find that the most common DDIs were generated from the combination of COVID-19 drugs with anti-infective agents compared with other kinds of drugs in patients. Meanwhile, it should be noted that four antiviral agents, ritonavir, darunavir, daclatasvir and sofosbuvir could interact with many drugs as indicated. Some immune regulators such as budesonide and prednisone also presented many efficacy-related DDIs. We found that DDIs that could increase the risks of potential toxicity events, were mainly linked with anti-septic shock agents and immune regulators, such as heparin, enalapril and colchicine. DDIs with drugs for other purposes are presented in the supplementary data (Fig. S1), and a summary of all of these DDIs can also be found in Table S3.

These results indicated that the DDIs of antiviral agents (ritonavir, darunavir, daclatasvir and sofosbuvir), immune regulators (budesonide, colchicine and prednisone), heparin and enalapril should be preferentially considered for potential side effects and efficacy of drug treatment.

### Polymorphic pharmacogenes in COVID-19 therapy

**Fig. 4:**
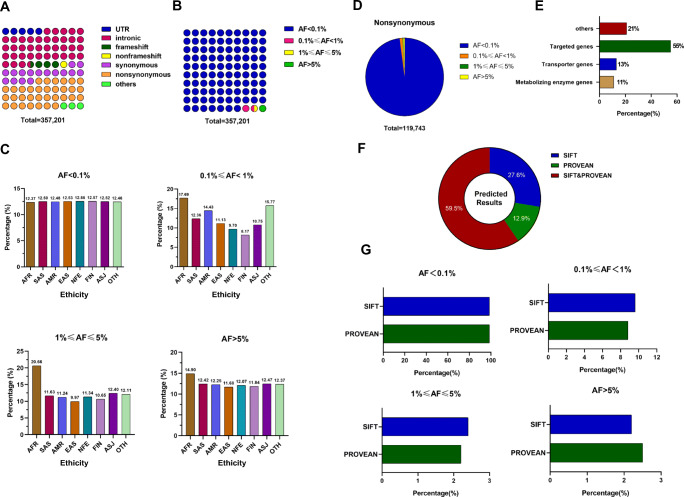
**Variation profiles of pharmacogenes.** (A) and (B) The distribution of all variations in pharmacogenes, which were classified according to their locations and functions (A), and MAF (B). The different colours of the circle indicate the different characteristics of variants in (A), or indicate the different MAFs in (B), while the number of circles indicates the percentage of a group of variations according to their colours as the legends indicate. (C) The proportion of variations with different MAFs in eight different ethnic groups, and the height of the column indicates the proportion in each ethnic group. (D) The distribution of nonsynonymous variations according to their MAF. The different colours of the sector indicate the different MAFs, and the proportion of the sector in the pie chart represented the percentage of a group of variations according to their colours as legends indicate. (E) The distribution of nonsynonymous variations according to the gene categories, including metabolizing enzyme genes, transporter genes, targeted genes and others. (F) The proportion of potentially damaging variations predicted by SIFT or PROVEAN software. (G) The distribution of potentially damaging variations, which were predicted by each software, according to their MAF. AFR-African, AMR-Latino, EAS-East Asian, SAS-South Asian, FIN-Finnish, NFE-non-Finnish European, ASJ-Ashkenazi Jewish, OTH-Other. UTR-untranslated regions, AF-allele frequency, SNV-single nucleotide variants, MAF-minor allele frequency

Genetic variation has been considered one of the most powerful biomarkers to guide personalized therapy [32, 33]. Thus, it is important to explore genetic variants in pharmacogenes that may affect drug response and toxicity [34, 35]. A total of 373 pharmacogenes related to all of these drugs used for COVID-19 treatment were retrieved (Table S4). Based on the analysis of 125,748 subjects, 357,201 variations in these pharmacogenes were found. Although mutations were distributed in all gene regions, the most common mutation types were intronic, nonsynonymous and synonymous mutations (Fig. 4A-E). It should be noteworthy that 33.52% of the mutations were nonsynonymous, which could potentially change the function of pharmacogenes. Such changes could potentially explain individual and ethnic differences in COVID-19 drug treatment. Although 98.50% of the mutations were rare genetic variants with minor allele frequencies (MAFs) lower than 1%, their cumulative effects should not be ignored.

To predict the significance of all nonsynonymous variations in these pharmacogenes, PROVEAN and SIFT tools were utilized. Their performances were similar in functional variation identification (Fig. 4F). A total of 72.4% of all these nonsynonymous variations were predicted by SIFT, while 87.1% were predicted by PROVEAN. The 59.5% of these potential functional nonsynonymous variations were predicted by both SIFT and PROVEAN, while 12.9% and 27.6% of these potential functional nonsynonymous variations were predicted by PROVEAN and SIFT only, respectively. The frequency distributions of these potential functional nonsynonymous variations which were predicted by these two tools were similar, too (Fig. 4G). These results indicated that these two tools were reliable, and most of these potential functional variations could be simultaneously predicted by both tools. All potential functional variants were next enrolled to calculate the “Gene Score”, a parameter that represented the cumulative functional variant carrier homozygote rate. Ninety genes carried a gene score above 0.01 among all 373 pharmacogenes with predicted functional nonsynonymous variations. These genes could carry at least 1 homozygous potentially deleterious allele in 1% of patients (Table S5). The higher “Gene Score” of these pharmacogenes again indicated potential individual differences for COVID-19 treatment, and we should pay more attention to these pharmacogenes. For example, the “Gene Score” of *G6PD* is 0.013 in AFR, which is higher than other ethnic groups (the second largest “Gene Score” is 0.002 in NFE). Therefore, abnormal *G6PD* should receive more attention in AFR than in the other seven ethnic groups as indicated, and drugs related to *G6PD* should also be more likely to generate individual differences in AFR, too (Table 2).

From these analyses, many potential functional variations were predicted in the pharmacogenes. These variations may affect the efficacy and toxicity of drugs in COVID-19 treatment, which indicates the importance of personalized drug therapy and PPPM strategies.

### Actionable pharmacogenes and their mutations

**Fig. 5:**
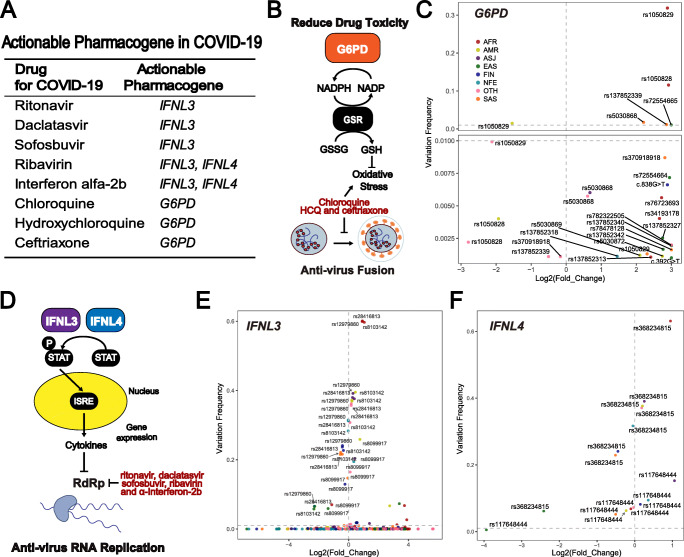
**Functional variations in actionable pharmacogenes.** (A) Eight actionable pharmacogene-drug pairs in COVID-19 treatment. (B) The mechanisms of *G6PD* in the occurrence of drug toxicity. The box with the gene name indicates the crucial pharmacogenes involved in the pathway. The arrow in the pathway indicates stimulation or conversion, while the “T”-type arrow indicates an inhabitation instead. (C) Frequency analyses for functional variants in *G6PD*. (D) The mechanisms of *IFNL3* and *IFNL4* in facilitating drug efficacy. The box with gene name indicates the crucial pharmacogenes involved in the pathway, and the box with a “P” mark on the upper left side indicates a phosphorylated protein. The arrow in the pathway indicates stimulation or conversion, while the “T”-type arrow indicates inhibition instead. The large yellow ellipse indicates the nucleus of cells, and the process in the ellipse indicated that this process occurred inside the cell nucleus and vice versa. (E) Frequency analyses for functional variants in *IFNL3*. (F) Frequency analyses for functional variants in *IFNL4*. For (C), (E) and (F), the Y-axis indicates the frequency of variations, while the X-axis indicates the fold change of variations. For fold change computation, the mean frequency of each functional variation among all populations was utilized as the standard. Different colours indicate different populations, as indicated in the legends. AFR-African, AMR-Latino, EAS-East Asian, SAS-South Asian, FIN-Finnish, NFE-non-Finnish European, ASJ-Ashkenazi Jewish, OTH-Other. *G6PD*-Glucose-6-phosphate dehydrogenase, *IFNL3*-interferon lambda 3, *IFNL4*-interferon lambda 4

Based on our analysis, some actionable PGx biomarkers could be used for COVID-19 treatment. There were eight drug-gene pairs that should be considered, including: chloroquine-*G6PD*, HCQ-*G6PD*, ceftriaxone-*G6PD*, ritonavir-*IFNL3*, daclatasvir-*IFNL3*, sofosbuvir-*IFNL3*, ribavirin-*IFNL3*/*IFNL4*, and interferon alpha 2b-*IFNL3*/*IFNL4* (Fig. 5A). Variants in these pharmacogenes could alter their function and affect their correlated drug efficacy or toxicity (Fig. 5B and D). We then performed analyses of the functional variants of *G6PD*, *IFNL3* and *IFNL4*.

For *G6PD*, a total of 109 functional variations were included for further analyses. All of these functional variations in *G6PD* were nonsynonymous variations, and the deleterious functions of these variations in *G6PD* were already validated by previous studies. However, except for five single nucleotide polymorphisms (SNPs), most of these variations showed very low mutation frequencies. The SNP rs1050829 had the highest frequency among all variations, and the frequency of this variation in the whole population was 31.86% (Fig. 5C). The second highest variation was rs1052828, and its frequency was 11.56%. However, the frequency of the third highest variation, which was recognized as rs5030868, was only 1.73%. In addition, the frequencies of rs137852339 and rs72554665 were higher than 1%. Among these variations, rs5030868 and rs72554665 can reduce the activity of *G6PD* from normal to less than 10%, while rs1050829, rs1052828 and rs137852339 can decrease it to 10% ~ 60% [36, 37]. These variations were clinically important because they can attenuate *G6PD* activity, thus indicating the risk of drug-induced acute haemolytic anaemia (Fig. 5B). The high frequency of these mutations indicated that their carriers had a higher probability of accidental toxicity events for drug utilization of chloroquine, HCQ and ceftriaxone.

*IFNL3* and *IFNL4* are two adjacent genes on chromosome 19. Fewer variations were reported in these two genes than in *G6PD*. In *IFNL3*, only four variants, rs8103142, rs12979860, rs8099917 and rs28416813 were reported (Fig. 5E). We then conducted systematic analyses of these important variants. Among these variations, rs8103142 was a nonsynonymous variation whose frequency in the whole population was 30.75%. This result indicated that almost one third of individuals carried this functional variation among all patients undergoing COVID-19 treatment. The rs28416813 was located in the 5’UTR of *IFNL3*, and the frequency of this allele was 60.08%, which is the highest frequency variation in *IFNL3*. rs12979860 and rs8099917 were two intronic variations, and the mechanisms of these two variants were not thoroughly clarified. The frequencies of rs12979860 and rs8099917 were 59.95% and 25.88% respectively. All four variations have been reported to impact drug effects in a large number of clinical trials involving antiviral regimens, including ritonavir, daclatasvir, sofosbuvir, ribavirin and interferon alpha 2b [38–40]. Therefore, rs8103142, rs12979860, rs8099917 and rs28416813 may also have a negative impact on the efficacy of these drugs in COVID-19. In *IFNL4*, only two variants, rs368234815 and rs117648444 were reported (Fig. 5F). As our results indicated, the frequency of rs368234815 was 63.09%, while that of rs117648444 was only 15.28% (Fig. 5F). These two variants were both not nonsynonymous variations, and were located in the intron of *IFNL4*.

In addition to the variations mentioned above, mutations with low frequencies (<0.1%) should also be considered. Such mutations constituted the major proportion and their cumulative effect should not be ignored, especially for *G6PD*. These variations are described in the supplementary data (Fig. S2). In addition, ethnic differences were analysed for these actionable pharmacogenes. We found that most functional variations of the three genes showed ethnic bias among populations. As indicated, Africans showed an obviously higher frequency than other populations for most of the mutations (Fig. 5C, E and F).

In summary, most of the variations in actionable pharmacogenes were low frequency mutations. Therefore, variations with higher frequency should be first considered. They were rs1050829, rs1052828, rs5030868, rs137852339, rs72554665 in *G6PD*, rs8103142, rs12979860, rs8099917, rs28416813 in *IFNL3*, and rs368234815, rs117648444 in *IFNL4*. In addition, ethnic differences in variations in particular pharmacogenes should also be considered.

### Analysis of drug effects in the context of ethnic differences

As we mentioned in Fig. 2C, many drugs were influenced by ethnicity-based differences in COVID-19 treatment. However, only limited actionable pharmacogenes were available in the current stage, although a large number of pharmacogenes showed ethnic disparities (Fig. 4C). It is obvious that pharmacogenetic studies should be conducted to find more actionable pharmacogenes to direct PPPM-attitude-based COVID-19 management, while the priority of drugs that should be studied has still not been systematically evaluated.

Generally, drugs influenced by ethnicity-based differences in pharmacogenes should be studied first. As mentioned above, the differences in trials among different ethnic groups were determined in 132 reported COVID-19 studies (Fig. 2C). To comprehensively explore the ethnicity-based differences in pharmacogenes of all drugs in COVID-19 therapy, we employed the “Drug Score”. These scores were calculated based on the cumulative “Gene Score” deviations of all pharmacogenes between one ethnicity and seven other ethnic groups for each drug and represented the ethnicity-based differences of this drug compared with seven other ethnic groups. For example, the “Drug Score” of remdesivir in EAS is 0.214, which is much higher than the second highest score (0.092 in FIN). This means that the frequencies of EAS functional variations in all pharmacogenes of remdesivir were much more different than those of the other seven ethnic groups, and indicates that the impacts of pharmacogenes on remdesivir could be much more different in EAS than in other ethnic groups. Therefore, pharmacogenetic studies for remdesivir could be important to explain the different therapeutic effects of remdesivir in EAS compared with other ethnic groups, and related PPPM-attitude-based COVID-19 management could be provided based on these studies.

**Fig. 6:**
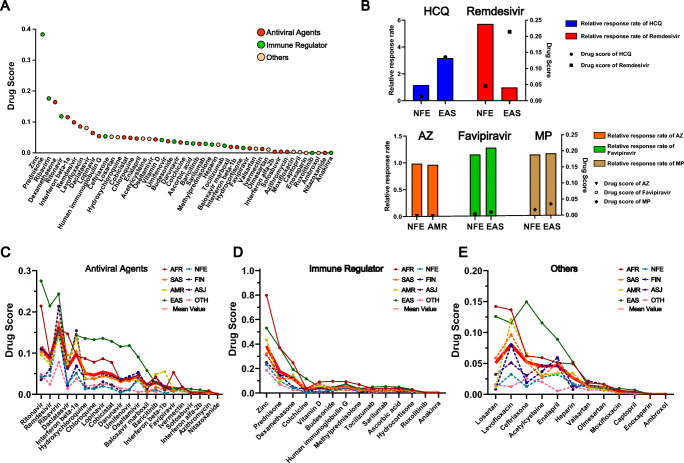
**Ethnicity-based differences in drug utilization in COVID-19 treatment.** (A) The mean “Drug Scores” calculated for all drugs in COVID-19 treatment. The red dots, green dots and orange dots indicate antiviral agents, immunomodulators and other drugs in the COVID-19 regimen respectively. (B) The results of clinical trials in different ethnic groups and relevant “Drug Scores” in these ethnic groups. The upper panel presents examples that showed different drug efficacies between the two ethnic groups, while lower panel presents examples that did not show different drug efficacies between the two ethnic groups. The height of the column indicates the relative response rate of a drug in an ethnic group, while the dot in the same lane indicates the “Drug Scores” of this drug in this ethnic group. (C-E) The ethnicity-based differences of “Drug Scores” calculated for (C) antiviral agents, (D) immune regulators, and (E) other categories of drugs in COVID-19 treatment. HCQ-hydroxychloroquine, AZ-azithromycin, MP-methylprednisolone. Fine lines with different colours indicate the “Drug Scores” in different ethnic groups, as the legends indicate, while the thick red line indicates the mean “Drug Scores” of all eight ethnic groups. AFR-African, AMR-Latino, EAS-East Asian, SAS-South Asian, FIN-Finnish, NFE-non-Finnish European, ASJ-Ashkenazi Jewish, OTH-Other

Then the “Drug Scores” of 47 drugs were calculated and ranked. As shown in Fig. 6A, they were quite different. Based on the available data, we analysed the five drugs in detail (Fig. 6B). For remdesivir, the “Drug Score” was very different between non-Finnish Europeans (0.05) and East Asians (0.21), and the relative survival rate in non-Finnish Europeans was 5.83 times higher than that in East Asians [19, 41]. For HCQ, the “Drug Scores” were 0.01 and 0.14 in non-Finnish Europeans and East Asians, respectively. Remdesivir can eliminate SARS-CoV-2 nucleic acid in East Asians but not in non-Finnish Europeans with viral clearance rates of 317.14% and 116.48%, respectively [42, 43]. The “Drug Scores” of azithromycin, favipiravir and methylprednisolone were relatively low. Correspondingly, the differences in clinical drug efficacy were also small. Therefore, the “Drug Score” could be used to screen drugs with large ethnicity-based pharmacogenetic differences in COVID-19.

We defined drugs with a “Drug Score” greater than 0.05 as significantly different among ethnic groups. As shown in Fig. 6A, these drugs included zinc, prednisone, ribavirin, dexamethasone, ritonavir, interferon beta-1a, remdesivir, levofloxacin, daclatasvir, lopinavir, human immunoglobulin G, ceftriaxone, losartan and HCQ. The ethnicity-based differences of these drugs need more attention when used in the treatment of COVID-19. Figure 6C-E further indicates the “Drug Scores” in different populations. These can be used to explain the high “Drug Scores” in more detail. Each of the abovementioned 14 drugs should be given more attention in specific populations. These are summarized in Table 3. These drugs were suggested to be given priority for conducting pharmacogenetic studies, and PPPM-attitude-based COVID-19 management could be facilitated according to the results of these studies.

## Discussion

This study highlighted potential interindividual differences including comorbid diseases and genetic backgrounds, as well as it highlighted our suggestion that PPPM attitude should be considered in the COVID -19 therapy procedures. Through DDI querying and genetic testing for actional pharmacogenes, patients could be treated with a lower risk of adverse effects and with higher health benefits. Moreover, our study focused on almost all frequently utilized drugs in COVID-19 treatment, while specific information such as detailed DDIs and variations in actionable pharmacogenes were thoroughly analysed. We also provide recommendations in the “Conclusions and Recommendations” section based on our results, which can facilitate translation of our results into routine hospital procedures or primary health care, offices of general practitioners. Moreover, PPPM-attitude-based COVID-19 management can reduce the possibility of additional interventions for serious DDIs or unfavourable alleles in actional pharmacogene-generated toxicity, thus improving the cost-benefit of COVID-19 treatment [44, 45].

Based on the current observations, the symptoms in COVID-19 patients are complicated [6, 7]. This clinical characteristic indicates that there is a very high possibility of drug combination during COVID-19 treatment. In addition, elderly individuals comprise consisted a large portion of COVID-19 patients, and the death rate of older patients was reported to be higher in COVID-19 therapy [46]. They may suffer from more diseases such as cancer, hypertension or diabetes before infection [47]. To control these symptoms, even more drugs may be used, which increases the complexity of drug treatment. Our results showed that there were at least 397 reported DDIs, which were recorded by the FDA, during COVID-19 drug treatment. Four antiviral agents (ritonavir, darunavir, daclatasvir and sofosbuvir) should be paid attention due to DDIs that can influence agents’ efficacy. For example, darunavir could increase the concentration of the *CYP3A*-metabolized narcotic analgesic fentanyl and lead to potentially fatal respiratory depression. Among them, ritonavir and darunavir interacted with 119 drugs respectively, which indicated that the two drugs should be used more carefully when treating patients with comorbidities. In contrast to antiviral agents, immune regulators, such as budesonide, colchicine and prednisone were more related with toxicity. For instance, coadministration of budesonide inhalation suspension with the cardiovascular drug ketoconazole may induce adverse effects related to increased systemic exposure to budesonide. Therefore, careful monitoring of therapeutic and adverse effects is recommended when these drugs are concomitantly administered with commonly utilized drugs.

PGx defines genetically-determined variability in drug response. Pharmacogenes are considered to be important genes affecting drug response. Their genetic variations contribute to drug responses or toxicity via pharmacodynamics or pharmacokinetics pathways. According to our results, 119,734 nonsynonymous variations in pharmacogenes related to COVID-19 drugs were found. This implied the large individual disparity of treatment. Most importantly, 3 actionable pharmacogenes were found to guide personalized treatment for COVID-19 with 8 drugs. These pharmacogenes, such as interferon-*IFNL3* have shown solid predictive performance of drug response in previous studies. For patients with hepatitis C treated with pegylated interferon-alpha, the *IFNL3* genotype is the strongest pretreatment predictor of drug response. For example, rs12979860 CC genotype patients received 23% ~ 30% more benefits than TT and CT genotype patients [48]. This result indicated that patients with different genotypes may present different responses to interferon-alpha therapy. Similarly, other variations (rs8103142, rs12979860, rs8099917 and rs28416813) of *IFNL3* were also reported to be associated with the response to pegylated interferon-alpha and ribavirin. Variants of *IFNL3* and its upstream gene *IFNL4* were strong predictors among the drugs (interferon-alpha, ribavirin, ritonavir, darunavir, daclatasvir and sofosbuvir) used in Hepatitis C virus (HCV) patients. These variations in *IFNL3* and *IFNL4* may have an impact on the efficacy of these drugs in COVID-19 treatment. *G6PD* variants rs5030868 and rs72554665 can reduce the activity of *G6PD* from normal to less than 10%, while rs1050829, rs1052828 and rs137852339 can decrease it to 10% ~ 60%. In *G6PD* deficient patients, acute haemolytic anaemia occurred with chloroquine and HCQ treatment, and methemoglobinemia was observed with ceftriaxone treatment. Therefore, it is reasonable to speculate that these mutations in *G6PD*, *IFNL3* and *IFNL4* could also affect the efficacy and serious side effects of paired drugs.

However, only limited actionable gene-drug pairs were utilized for PPPM in the current stage. One of the main factors is the high cost of conducting a well-designed PGx trials for personalized medicine. To identify a solid biomarker, recruitment of a large sample size and a complex composition of patients with different genetic backgrounds is essential. Another factor is that the benefit the patients can obtain from PGx testing should be properly weighted during the implementation of a clinical guideline for personalized medicine [49]. For example, different ethnic groups, that carry functional variants with different allele frequencies, may benefit more or less from the same PGx testing. This means that large scale pharmacoeconomic studies are also important in the promotion of personalized medicine. Although these factors are obstacles against personalized drug treatment in clinical practice, they can be solved if more clinical trials will be conducted in the future. Thus, there may be more PGx biomarkers that need to be confirmed and validated by clinical trials, and they could predict drug responses and provide PPPM with a powerful tool for COVID-19 management and treatment.

In addition, one of the most significant challenges in COVID-19 treatment is that SARS-CoV-2 has spread widely all over the world. Among global clinical trials, the responses of drugs to COVID-19 have varied (Fig. 2C), and the ethnicity-based differences of clinical trials have caused widespread public concern and controversy. These paradoxical outcomes may be caused by ethnicity-based differences in pharmacogenes as we discussed above. For example, remdesivir showed controversial clinical trial outcomes between Chinese and Americans in COVID-19 treatment, and the ethnic differences in pharmacogenes related to remdesivir in these two countries may be one reason, as we reported [48, 50]. In this study, we calculated the cumulative effects of functional variants in pharmacogenes, systematically assessed the ethnicity-based differences of 47 drugs utilized in COVID-19, and then verified our assessment results by comparing them with the reported outcomes of COVID-19 clinical trials. The outcomes of clinical trials were consistent with our assessment, and some drugs, like remdesivir and HCQ, showed high differences related to the ethnic origin of the patients and presented different responses in different ethnic groups in COVID-19 treatment. Other drugs, such as azithromycin, favipiravir and methylprednisolone, with “low ethnic differences” showed the same response to treatment. Although vaccines for preventing COVID-19 are widely implemented worldwide, the individual disparities in COVID-19 vaccines have also been reported. Our strategies for DDI analysis could also be useful to predict DDIs for COVID-19 vaccines thus preventing patients from suffering from failed protection or serious side effects based on their personalized characteristics. Therefore, we propose that the PPPM-related suggestions presented in our study can be utilized to solve similar, potentially DDI-related, problems in relation to vaccines, and help to manage current COVID-19 or other outbreaks in the future. All of these results indicated that individual differences could explain discrepancies in the outcomes of COVID-19 clinical trials. We believe that more drugs should be “calculated” and explained using our attitude in the future to increase the benefits of drug therapy while substantially decreasing the risk of negative side effects for the patients.

## Strength and limitations

In this study, DDIs and actionable pharmacogenes were systematically analysed to promote PPPM-attitude-based COVID-19 management. As our results indicated, patients with different characteristics such as basal complication status and genetic background should be distinguished, and personalized COVID-19 regimens should be prescribed based on these characteristics. We also provide an example here of what may happen to a patient if they or medical personnel, do not follow the PPPM-based attitude in COVID-19 treatment. If a diabetic patient is diagnosed with COVID-19 and is prescribed HCQ, a fatal risk may be generated if this patient is using metformin concomitantly [51]. Moreover, if this patient is a deficient G6PD allele carrier, severe haemolysis crisis can occur under the utilization of HCQ [52]. These risks can be largely decreased if the DDI evaluations or genetic tests are provided for clinicians or patients. Therefore, only PPPM attitude is highly recommended in COVID-19 management, and patients can benefit from being treated with the lowest risk of adverse effects and the highest health benefits.

The major limitation of this study is that more clinical trial data should be added to support our results. Although high quality data were included in this study, only preliminary results supporting personalized medicine in COVID-19 treatment were presented. More preclinical studies and clinical trials are certainly needed to explore individual disparities in these drugs. Moreover, genetic information of individuals from 8 ethnic groups was acquired from the GnomAD project, which is the largest human genome project of different ethnicities to data (Table S6). Therefore, these 8 ethnic groups were also classified based on the rules established by this project [53]. Other ethnic groups that are also important should not be ignored, although they were not studied in this study. Meanwhile, the clinical practice of general practitioners or hospital/specialized medical centres from the point of view of practical implementation may not be easy, as genetic testing may be difficult to perform in some regions where people/patients carry a risk allele. We therefore suggest that these patients should avoid using high-risk drugs if reliable genetic tests are still not available. Other factors such as gender and age of the patient were also important in managing personalized COVID-19 treatment and should not be overlooked by clinicians. However, we did not analyse the impact of these factors on the personalized COVID-19 management due to the limited data, so that further studies about these factors are suggested in the future.

## Conclusions and recommendations

In this study, 47 drugs for COVID-19 treatment were analysed. We focused on three aspects including DDI, variations in pharmacogenes and the differences in drug utility arising from the ethnic differences. We suggest that 22 drugs should be taken into consideration for PPPM. Among them, we recommended that antiviral agents (ritonavir, darunavir, daclatasvir and sofosbuvir), immune regulators (budesonide, colchicine and prednisone), heparin and enalapril which could generate the most DDIs with commonly concomitantly utilized drugs should be under higher supervision to avoid potential DDIs. We recommended that genetic testing for particular SNPs in specific genes should be utilized for 8 drugs (ritonavir, daclatasvir, sofosbuvir, ribavirin, interferon alpha-2b, chloroquine, HCQ and ceftriaxone) that have actionable PGx biomarkers especially in ethnic groups with higher frequency functional variations as our analysis found. We also propose that pharmacogenetic studies of 14 drugs (ritonavir, daclatasvir, prednisone, dexamethasone, ribavirin, HCQ, ceftriaxone, zinc, interferon beta-1a, remdesivir, levofloxacin, lopinavir, human immunoglobulin G and losartan) that showed significant ethnic genetic differences should be considered for further PPPM development and practical application.

**Fig. 7:**
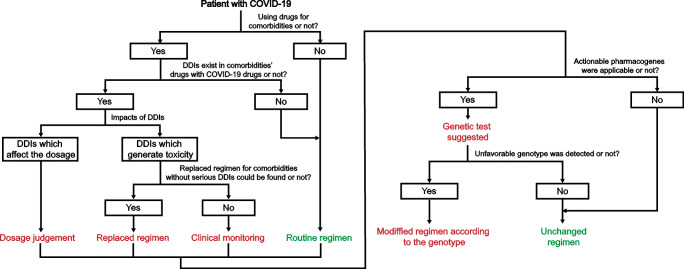
**A suggested PPPM process for facilitating the implementation of our results in routine clinical practice.** A detailed process was provided for potential COVID-19 patients if our results could be implemented in clinical practice. Choices and final recommendations for COVID-19 regimen determination according to the personalized characteristics of each patient for all possible situations are provided in a step-by-step process description

As stated above, we conclude that our study could be helpful in routine clinical practice for COVID-19 patients if suitable guidelines are implemented. For this purpose, we also provide an example of how to apply our results to guide drug utilization in COVID-19 patients using a PPPM attitude (Fig. 7). We suggest that when a patient is diagnosed with COVID-19, the first step is to determine whether the patient is using drugs for comorbidities. If not, a routine regimen for COVID-19 can be considered. In contrast, a potential COVID-19 treatment regimen that will not generate DDIs should be considered the first choice (all DDIs could be searched in the Table S2). If the DDIs of the COVID-19 regimen and the patient’s basic regimen cannot be avoided, the patient or the medical staff can consider adjusting the dosage if the DDIs only impact the concentration of therapeutic agents. Alternately, the medical staff or the patient should try to replace the patient’s basic regimen to avoid potential DDIs that could cause toxicity or serious side effects. If there is no better choice for a patient to avoid the serious DDIs of the COVID-19 regimen and the patient’s basic regimen, a personalized clinical monitoring for upcoming DDIs is recommended for this patient. Once the regimen for COVID-19 is determined through the above process, we suggest a genetic test if actionable pharmacogenes are found in the COVID-19 regimen (all actionable pharmacogenes can be searched in Fig. 5A). Once an unfavourable genotype for the current COVID-19 regimen is detected in this patient, his/her COVID-19 regimen can be modified according to the genotype. Based on evidence showing that all circumstances can occur in every patient as we mentioned above, we suggest a PPPM attitude in routine COVID-19 management.

The results reported in this study are also in a good agreement with the overall PPPM attitude, as presented in publications by the European Association for Predictive, Preventive, and Personalized Medicine (EPMA) [54]. In agreement with EPMA strategies, evaluation of drug effects related to interindividual differences, including comorbid diseases and genetic backgrounds referred to in this study, are important to avoid failed COVID-19 treatment or avoidable adverse events. To provide greater possibility of PPPM/3 PM for personalized treatment, we also suggest that future studies focused on predicting negative side effects in particular individuals on the basis of the pharmacogenome analysis and known DDIs, should be conducted. Therefore, analysing DDIs and using the acquired knowledge to personalize treatment from the pharmacogenes perspective in the PPPM/3 PM context is, in our opinion, an essential component of coping with the COVID-19 outbreak. In the wider context of future healthcare this attitude should be applied for any future outbreaks as well as for therapeutic procedures in general.

## Supplementary Information

Figure S1: **Drug-to-Drug interaction profiling in the FDA.** The drug-drug interactions of DDIs in the FDA between COVID-19 treatment drugs and other drugs that did not belong to any of the categories in Figure 3. As legends indicated, the circles indicated drugs in the COVID-19 regimens, while the diamonds indicated drugs in the basic regimens. Among them, the red circles, the green circles and the orange circles were antiviral agents, immunomodulators and other drugs in the COVID-19 regimens respectively. Similarly, the grey diamonds represented other drugs the basic regimens. If there was a DDI between two drugs, these two drugs were linked by a line. The red line indicates that this DDI could generate toxicity, while the green line indicates that this DDI could impact the efficacy. (PDF 1632 kb)

Figure S2: **Functional variations in G6PD among different ethnic groups.** Frequency and comparison analyses for functional variants in G6PD were analysed in eight ethnic groups. Variants with rsID were reported in the CPIC guidelines or PharmGKB database. AFR-African, AMR-Latino, EAS-East Asian, SAS-South Asian, FIN-Finnish, NFE-non-Finnish European, ASJ-Ashkenazi Jewish, OTH-Other. (PDF 899 kb)

ESM 1 (XLSX 17 kb)

ESM 2 (XLSX 1470 kb)

ESM 3 (XLSX 12 kb)

ESM 4 (XLSX 14 kb)

ESM 5 (XLSX 63 kb)

ESM 6 (XLSX 11 kb)

## Data Availability

Drugs in COVID-19 treatment and related clinical trials results were collected from publications (PubMed: https://pubmed.ncbi.nlm.nih.gov/; MedRxiv: https://www.medrxiv.org/; BioRxiv: https://www.biorxiv.org/; Cochrane Library: https://www.cochranelibrary.com/;) and ClinicalTrials (https://www.clinicaltrials.gov/). Validated DDIs was retrieved from FDA (https://www.fda.gov/) and predicted DDIs were derived from DrugBank (https://www.drugbank.ca/). ATC codes were retrived from WHO collaborating centre (https://www.whocc.no/atc_ddd_index/). Pharmacogenes were obtained from PharmGKB (https://www.pharmgkb.org/) and DrugBank (https://www.drugbank.ca/) databases. Actionable PGx biomarkers were gathered from the FDA (https://www.fda.gov/drugs/science-and-research-drugs/table-pharmacogenomic-biomarkers-drug-labeling) and CPIC guidelines (https://cpicpgx.org/guidelines/). Reported functional variations were collected from CPIC guidelines (https://cpicpgx.org/guidelines/) and PharmGKB (https://www.pharmgkb.org/). All genetic variation data including allele frequency can be accessed in Genome Aggregation Database (http://gnomAD.broadinstitute.org/, version: 2.1.1).

## Code availability

All genetic variations were annotated by allele frequency, location and function in different populations using ANNOVAR (version: 2019Oct24) [23]. Functional mutations were predicted by PROVEAN and SIFT [24]. The drug-to-drug interaction network was constructed by Cytoscape software (version:3.7.1) [22]. All statistical analyses were performed by SPSS software (version: 18.0), and results with *p* < 0.05 were considered as statistically significant. Data visualization were perfomed by GraphPad Prism software (version: 8.0.2).

## Abbreviations

ACE2: Angiotensin converting enzyme 2
AFR: African
AMR: Latino
ARDS: Acute respiratory distress syndrome
ASJ: Ashkenazi Jewish
COVID-19: Coronavirus disease 2019
CYP3A: Cytochrome P450, family 3, subfamily A
CYP2C9: Cytochrome P450 family 2 subfamily C member 9
DDIs: Drug-to-drug interactions. These DDIs could impact on side effects and efficacy of COVID-19 treatments when co-administration of some drugs
EAS: East Asian
FIN: Finnish
G6PD: Glucose-6-phosphate dehydrogenase
HCQ: Hydroxychloroquine
HCV: Hepatitis C virus
IFNL3: Interferon lambda 3
IFNL4: Interferon lambda 4
LPV-r: Lopinavir-ritonavir
MAF: Minor allele frequency
NFE: non-Finnish European
OTH: Other
PGx: Pharmacogenomics
PPPM/3 PM: Predictive, preventive, and personalized medicine
RdRp: RNA-dependent RNA polymerase
SARS-CoV-2: Severe acute respiratory syndrome coronavirus 2
SAS: South Asian
SNPs: Single nucleotide polymorphisms
TMPRSS2: Transmembrane serine protease 2
WHO: World Health Organization
5’UTR: 5′ untranslated regions
Pharmacogene: Pharmacogene is any gene that interacts with a pharmaceutical substance or drug, which may influence the efficiency or toxicity of drugs in COVID-19 treatments
