# Supplementary figures and images for: Complex analysis of the personalized pharmacotherapy in the management of COVID-19 patients and suggestions for applications of predictive, preventive, and personalized medicine attitude
Source: EPMA J. 2021 Jul 16;12(3):307–24. doi: 10.1007/s13167-021-00247-0 (PMC8283099; doi:10.1007/s13167-021-00247-0)

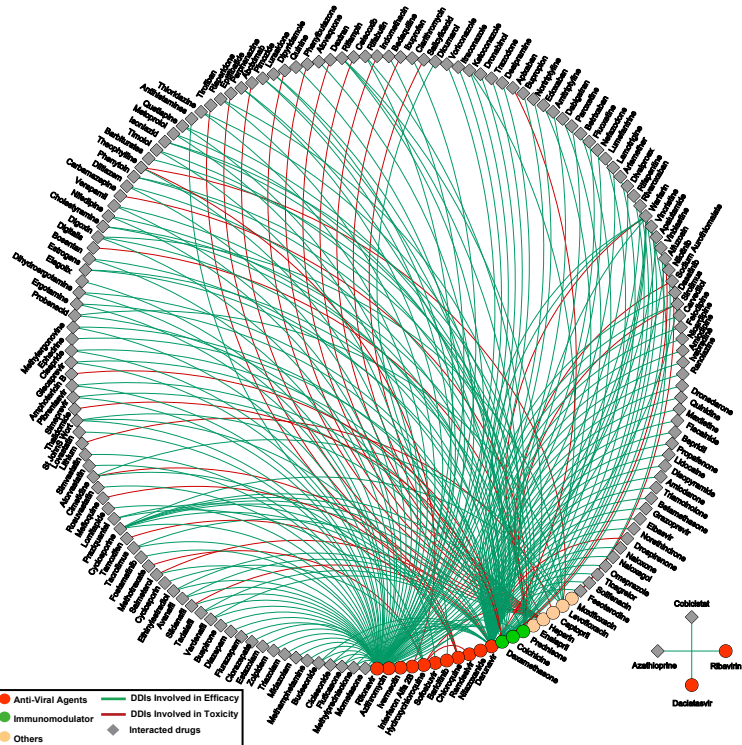

Supplement: Supplementary file 1 — Drug-to-Drug interaction profiling in the FDA. The drug-drug interactions of DDIs in the FDA between COVID-19 treatment drugs and other drugs that did not belong to any of the categories in Figure 3. As legends indicated, the circles indicated drugs in the COVID-19 regimens, while the diamonds indicated drugs in the basic regimens. Among them, the red circles, the green circles and the orange circles were antiviral agents, immunomodulators and other drugs in the COVID-19 regimens respectively. Similarly, the grey diamonds represented other drugs the basic regimens. If there was a DDI between two drugs, these two drugs were linked by a line. The red line indicates that this DDI could generate toxicity, while the green line indicates that this DDI could impact the efficacy. (PDF 1632 kb) [file 13167_2021_247_MOESM1_ESM.pdf]

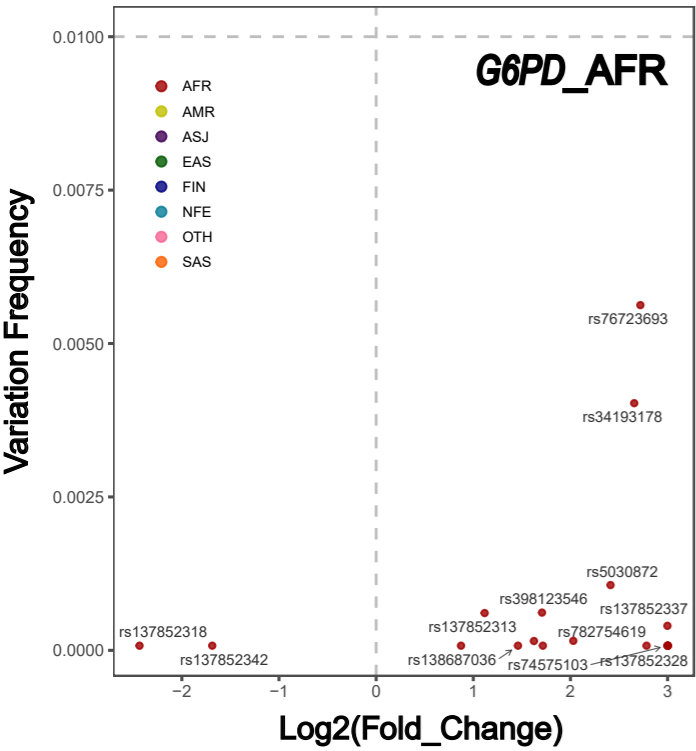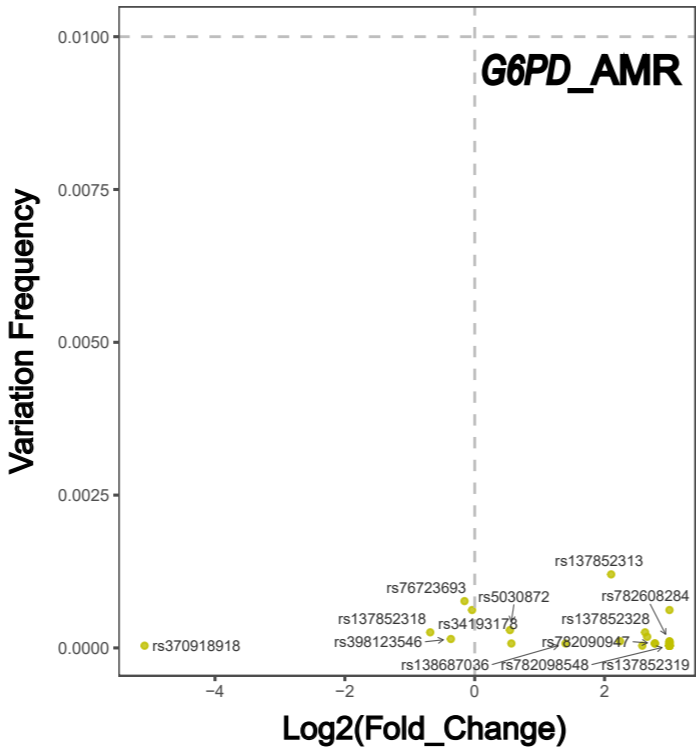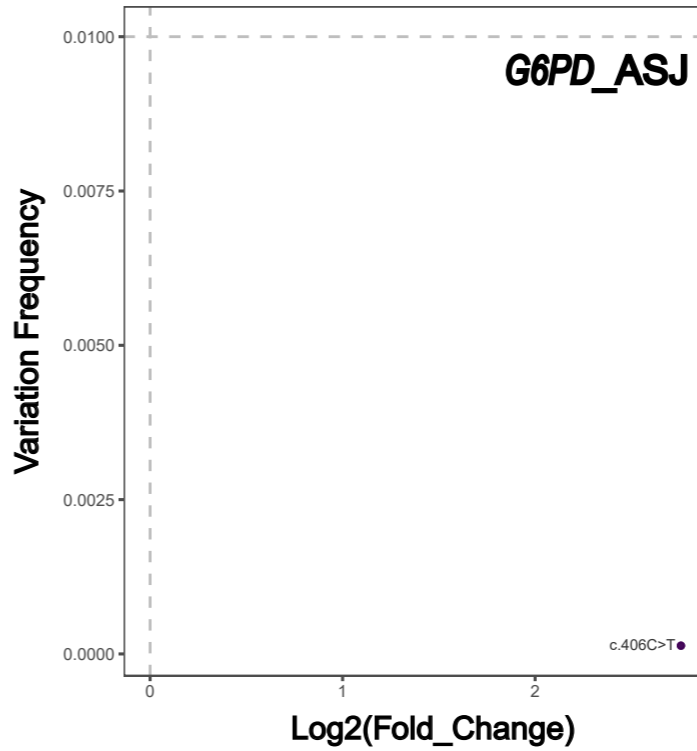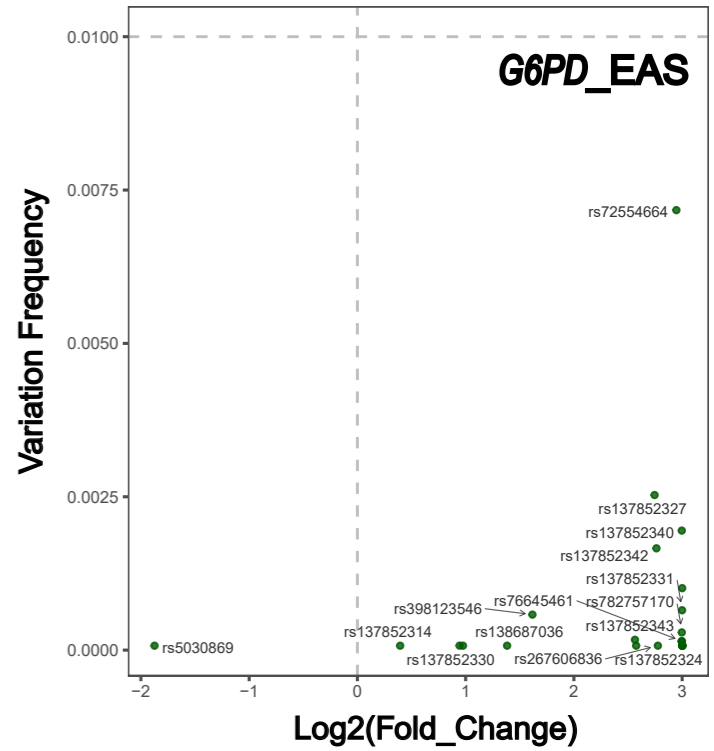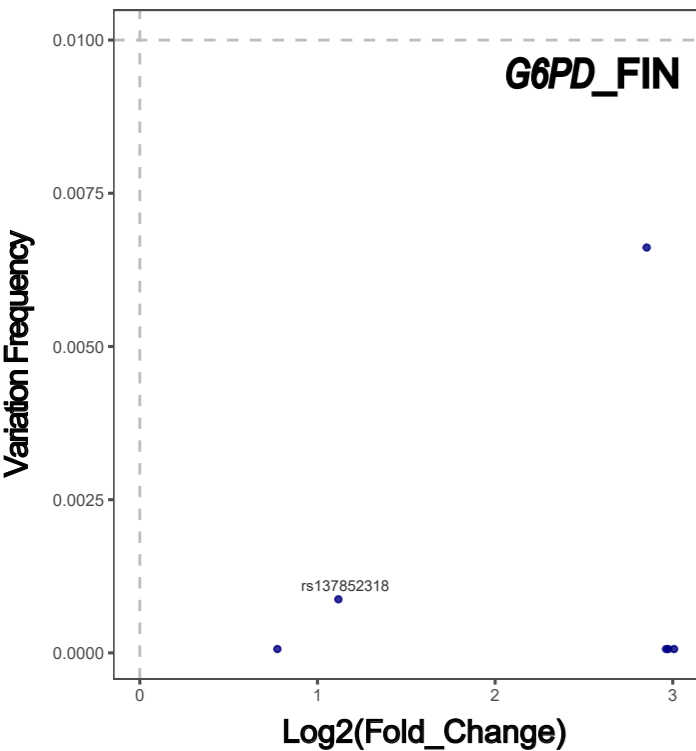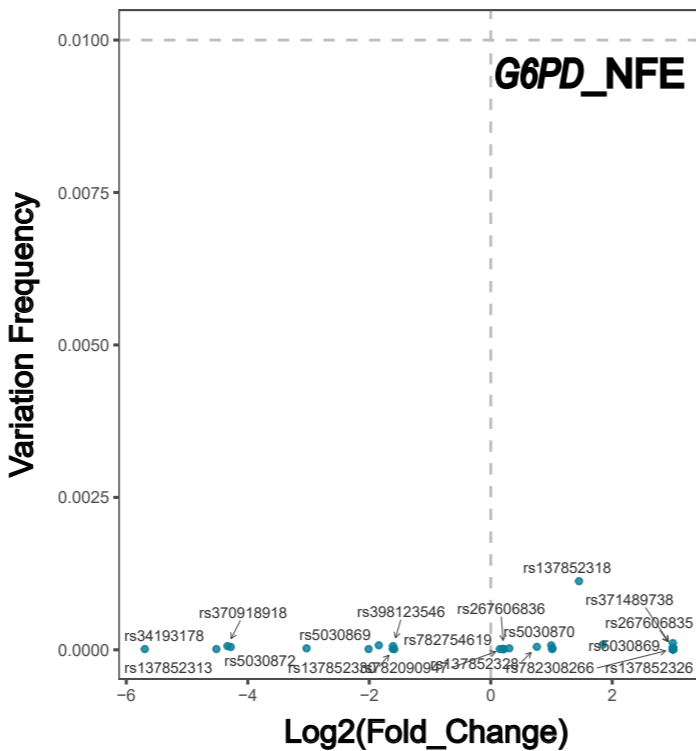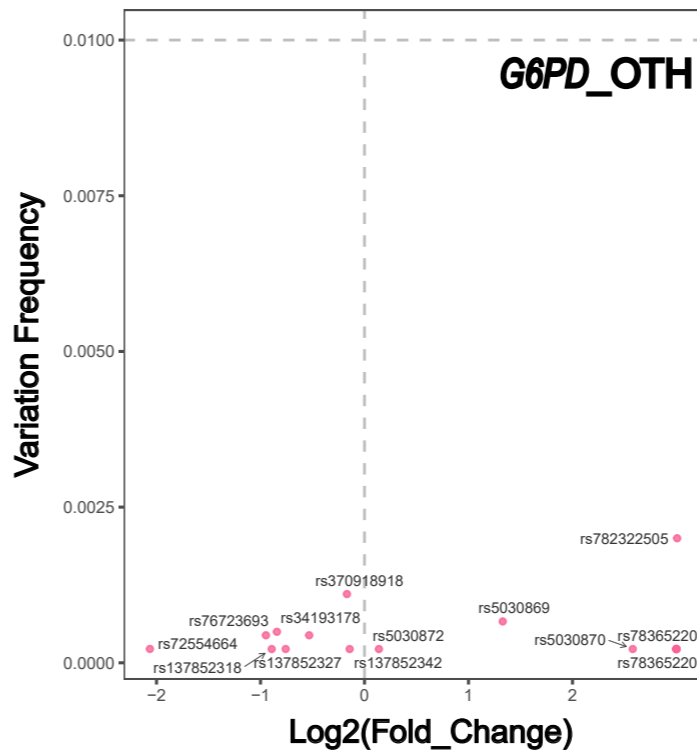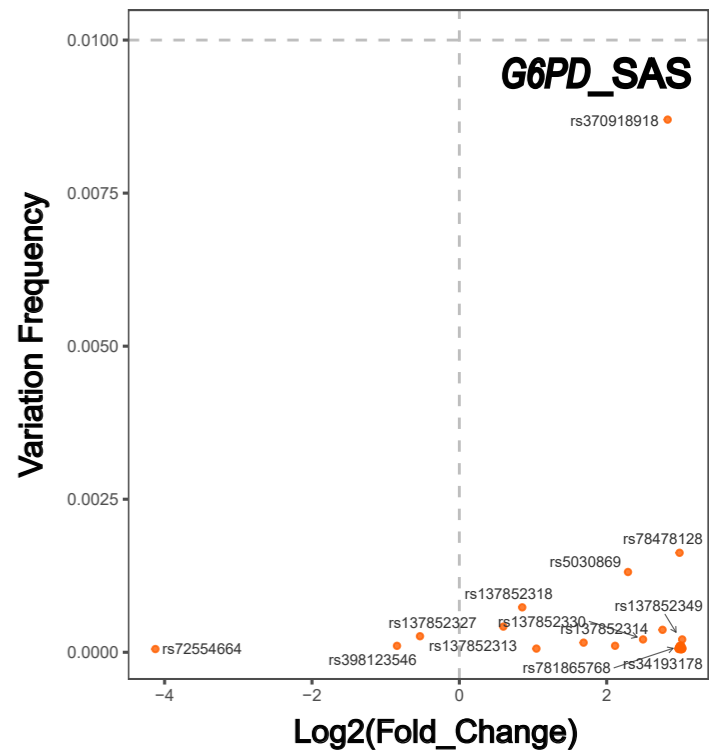

Supplement: Supplementary file 2 — Functional variations in G6PD among different ethnic groups. Frequency and comparison analyses for functional variants in G6PD were analysed in eight ethnic groups. Variants with rsID were reported in the CPIC guidelines or PharmGKB database. AFR-African, AMR-Latino, EAS-East Asian, SAS-South Asian, FIN-Finnish, NFE-non-Finnish European, ASJ-Ashkenazi Jewish, OTH-Other. (PDF 899 kb) [file 13167_2021_247_MOESM2_ESM.pdf]
